# miRNA‐21 deficiency impairs alveolar socket healing in mice

**DOI:** 10.1002/JPER.19-0567

**Published:** 2020-06-20

**Authors:** Franz Josef Strauss, Alexandra Stähli, Reiko Kobatake, Stefan Tangl, Patrick Heimel, Karol Alí Apaza Alccayhuaman, Markus Schosserer, Matthias Hackl, Johannes Grillari, Reinhard Gruber

**Affiliations:** ^1^ Department of Oral Biology Medical University of Vienna Vienna Vienna Austria; ^2^ Department of Conservative Dentistry School of Dentistry University of Chile Santiago Chile; ^3^ Clinic of Reconstructive Dentistry Center of Dental Medicine University of Zurich Zurich Switzerland; ^4^ Department of Periodontology School of Dental Medicine University of Bern Bern Switzerland; ^5^ Department of Advanced Prosthodontics Hiroshima University Graduate School of Biomedical and Health Sciences Hiroshima Hiroshima Japan; ^6^ Karl Donath Laboratory for Hard Tissue and Biomaterial Research Division of Oral Surgery School of Dentistry Medical University of Vienna Wein Wein Austria; ^7^ Austrian Cluster for Tissue Regeneration Medical University of Vienna Vienna Austria; ^8^ Ludwig Boltzmann Institute for Experimental and Clinical Traumatology Vienna Austria; ^9^ Institute of Molecular Biotechnology University of Natural Resources and Life Sciences Vienna Vienna Austria; ^10^ TAmiRNA GmbH Vienna Austria

**Keywords:** bone regeneration, mice, microRNA, mir‐21, tooth extraction

## Abstract

**Background:**

MicroRNAs (miRNAs) are small noncoding RNAs demonstrated as critical post‐transcriptional modulators in dental tissues and bone regeneration, particularly miR‐21‐5p. However, the role of miR‐21‐5p in the healing of alveolar sockets following tooth extraction remains unknown. In this study we evaluated the influence of miR‐21‐5p in the healing of alveolar socket after tooth extraction.

**Methods:**

Eight miR‐21‐5p knockout mice and eight littermate controls underwent tooth extraction of the upper right incisor. After a healing period of 14 days microCT and histological analyses were performed.

**Results:**

MicroCT analysis showed that the percentage of bone in the extraction socket was significantly higher in the control group than in the miR‐21 knockout mice; either in the coronal (39.0%, CI 31.8 to 48.0 versus 23.0%, CI 17.8 to 35.2, *P* = 0.03) or in the middle part of the alveolar socket (56.0%, CI 50.9 to 62.5 versus 43.5% CI 28.6 to 54.6, *P* = 0.03). These differences were not noted in the apical part of the extraction socket. Histological analysis supported the microCT findings. Newly bone volume per tissue volume (BV/TV) was significantly higher in the control group when compared to miR‐21 knockout mice, 27.4% (CI 20.6 to 32.9) versus 19.0% (CI 14.7 to 21.5, *P* < 0.05), respectively. Surprisingly, no evident signs of buccal bone resorption were observed in both groups.

**Conclusion:**

Despite the limitation of one observation period, these findings suggest that miR‐21‐5p delays the early healing of alveolar socket following tooth extraction. Whether miR‐21‐5p is essential for healing of alveolar sockets remains to be elucidated.

## 1 INTRODUCTION

Tooth extraction is a common procedure that generates an empty space in the alveolar socket. Following tooth extraction a cascade of conserved cellular events is triggered that culminate in the formation of bone at sites previously occupied by the tooth.^1^ This bone formation follows the principles of intramembranous ossification where mesenchymal cells, supplied by the blood sprouting capillaries, become bone‐forming osteoblasts.^2^ This sequence of events has been extensively investigated to further elucidate the process of fracture healing driven by the burden of the patients suffering from non‐healing and large size fractures.^3^, ^4^ In addition, there is a clinical demand to understand the healing of extraction sockets in dentistry mainly because of the rising need to restore the original tooth with dental implants.^5^ Because implant osseointegration in fresh extraction sockets follows the same principles of intramembranous ossification^6^ a better understanding of the healing of extraction sockets is desirable to improve implant‐based therapies.

Research on alveolar bone healing after tooth extraction is mainly based on large preclinical models that have provided insight into the conserved post‐extraction healing sequence.^1^ This sequence triggered by the tooth extraction induces the formation of a blood clot. Subsequently, this blood clot is organized into a connective tissue matrix that is later reinforced by woven bone and finally replaced by organized lamellar bone.^1^ This sequence of events also occurs when dental implants are placed into the alveolar bone as documented in canine models. The healing of tooth extraction has also been studied in rodent models, particularly in molar teeth of rats.^7^ It should be mentioned however, that rat models are reliable in simulating osteoporosis and diabetes but are not suitable when studying the impact of certain genes on the healing of the alveolar socket. Therefore, mice models were introduced by Vieira et al.^8^ to test the effect of certain genes on socket healing.^9^ Based on this concept, it was demonstrated that knockout of CD24 impairs bone healing following tooth extraction,^10^ that CBX7 deficiency improves bone healing in the alveolar sockets ^11^ and that CCR2 has no effect in bone healing.^9^ Consequently, this model has become increasingly used to evaluate the impact of particular genes on the healing of the extraction socket.

MicroRNAs (miRNAs) are small noncoding RNAs demonstrated as critical posttranscriptional modulators in dental tissues and are involved in tooth eruption and movement, differentiation of dental cells, and enamel mineralization.^12^ Although microRNA‐21‐5p (miR‐21‐5p) knockout mice seem to have no major skeletal phenotype,^13^ miR‐21‐5p deficiency prevents bone loss in mice by the inhibition of osteoclast.^14^ Similarly, mice lacking miR‐21 have compromised orthodontic tooth movement.^15^ However, the impact of miR‐21‐5p loss on alveolar socket healing still remains unknown. What is known is that high miR‐21‐5p levels accelerate fracture healing in rats^16^ and promote maxillofacial bone regeneration.^17^ Transient knockdown and overexpression of miR‐21‐5p are reducing and enhancing wound healing features respectively in mice.^18^ In humans, miR‐21‐5p is high in the circulation of osteoporotic patients,^19^ osteoporotic type 2 diabetes patients^20^ and osteoporotic fracture patients,^19^ and overexpression of it leads to lower osteogenic differentiation.^21^ The above summarized data give reason to suggest that miR‐21‐5p is a crucial element in the healing of extraction sockets. To test this hypothesis, we took advantage of mice lacking miR‐21‐5p and evaluated alveolar socket healing using the incisor extraction model.^8^ Here we indeed show that miR‐21‐5p is required for proper healing of the alveolar socket in vivo.

## 2 MATERIALS AND METHODS

### 2.1 Study design

The Medical University of Vienna ethical review board for animal research approved the study protocol (GZ BMWFW‐66.009/0080‐WF/V/3b/2017). The study was performed at the Department of Biomedical Research of the Medical University of Vienna in accordance with the ARRIVE guidelines.^22^ miR‐21 knockout mice were crossed into the C57BL/6J background and bred by mating of animals heterozygous for the miR‐21 knockout allele under specific‐pathogen‐free (SPF) conditions. Nine miR‐21 knockout mice and nine littermates (WT) controls (20 to 45 weeks, 20 to 25 g) underwent tooth extraction of the upper right incisor. The animals were treated according to the guidelines for animal care with free access to water and a standard diet.^23^

### 2.2 Tooth extraction model

FJS and AS performed the surgeries as previously described with some modifications.^8^ All animals received ketamine 100 mg/kg* and xylazine hydrochloride 5 mg/kg† by intramuscular injection. First, the head of the mouse was stabilized by holding the contralateral tooth with a tweezer. Next, with the aid of a stereomicroscope‡ under 16× magnification, the upper right incisor was luxated using disposable needles§ of different diameters (0.4 mm, 0.6 mm, and 0.8 mm) as periotomes (see Supplementary Video in online *Journal of Periodontology*). Then, the tooth was gently extracted to avoid any root fracture using an Adson tweezer¶ and checked for integrity. For pain relief, buprenorphine 0.06 mg/kg s.c.# and piritramide in drinking water ad lib was administered. The first 72 hours after surgery soft diet was provided. Mice were euthanized on day fourteen with an overdose of sodium pentobarbital at 300 mg/kg i.p. and each alveolar socket was subjected to micro computed tomographic (µCT) and histological analysis.

### 2.3 MicroCT analysis

**FIGURE 1:**
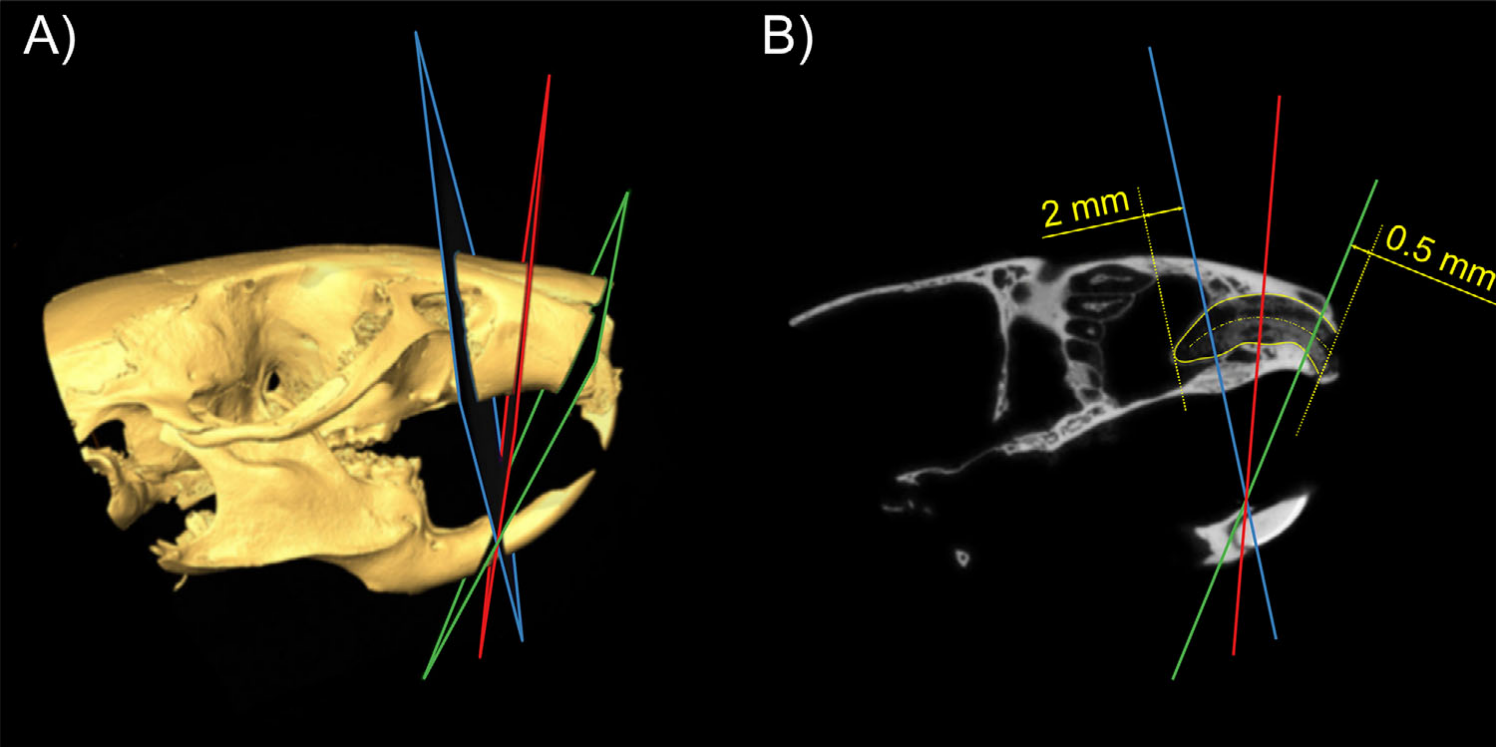
Positioning of the measurement slices in microCT data. The coronal (green), central (red) and apical (blue) oblique slices approximately perpendicular to the alveolar socket. The coronal slice was positioned at 0.5 mm from the most coronal part of the alveolar socket whereas the apical slice was positioned at 2 mm from the most apical part of the alveolar socket. The central slice was located approximately in the middle of both abovementioned slices. (**A**) Isosurface of the skull, (**B**) oblique slice oriented along the central alveolar socket (yellow)

After euthanasia the heads were fixed in phosphate‐buffered formalin.∥ *Micro*CT scans were made using a Scanco µCT 50** at 90 kV/200 µA with an isotropic resolution of 17.2 µm and an integration time of 500 ms. Using Amira 6.1.1,†† an oblique slice was positioned along the central axis of the alveolar socket. Perpendicular to this slice and approximately perpendicular to the central axis, three oblique slices were positioned in the coronal part, the middle and the apical part of the alveolar socket (Figure 1A and 1B). The regions were exported as images using the extract image tool. The extracted images were imported into Fiji.^24^, ^25^ The region of interest (ROI) was drawn using the polygon and freehand selection tools and saved using the ROI manager. Bone volume per tissue volume (BV/TV) was measured in the ROI using the bone volume fraction tools of the BoneJ plugin^26^ with a threshold of 254 mgHA/cm³. The thickness of the buccal bone plate was measured at four equidistant points using the coronal cross section slice through the root of the tooth and the alveolar socket for all the samples.

### 2.4 Histological and histomorphometric analysis

All samples were dehydrated with ascending alcohol grades and embedded in light‐curing resin‡‡. Blocks were further processed using Exakt cutting and grinding equipment.¶¶ Thin‐ground sections from all samples were prepared,^27^ in a plane parallel to the sagittal suture and through the middle of the alveolar socket and stained with Levai–Laczko dye. The slices of around 100 µm were scanned using an Olympus BX61VS digital virtual microscopy system§§ with a 20× objective resulting in a resolution of 0.32 µm per pixel and then evaluated. The region of interest was estimated to be the middle of the tooth extraction socket in an apico‐coronal direction, 1 mm in width, inside the alveolar plates. A rule set for the histomorphometry software Definiens Developer XD 2.7## was constructed to segment the mineralized bone tissue. The inaccurately segmented areas were manually corrected under visual control using Adobe Photoshop CS6.*** The following parameters were calculated on the whole region of interest: percentage of newly‐formed bone (nBV/TV) and the trabecular thickness (Tb.Th). The trabecular thickness was measured using Fiji using the BoneJ plugin on the segmented images. The bone close to the buccal and lingual cortical plates of the ROI was manually excluded from measurement.

### 2.5 Statistical analysis

Statistical analysis were based on the data observed with the microCT analysis. Median values and confidence intervals (CI) of the primary endpoint, bone volume per tissue volume (BV/TV) in the alveolar socket, between the two groups were compared with Mann‐Whitney *U* test. Secondary endpoints were also compared with Mann‐Whitney *U* test. Analyses were performed using Prism v7.* Significance was set at *P* < 0.05. Owing to the pilot nature of the study, the sample size was chosen based on experience from previous studies^8^ to balance the ability to measure significant differences while reducing the number of animals used.

## 3 RESULTS

### 3.1 Micro CT analysis

**FIGURE 2:**
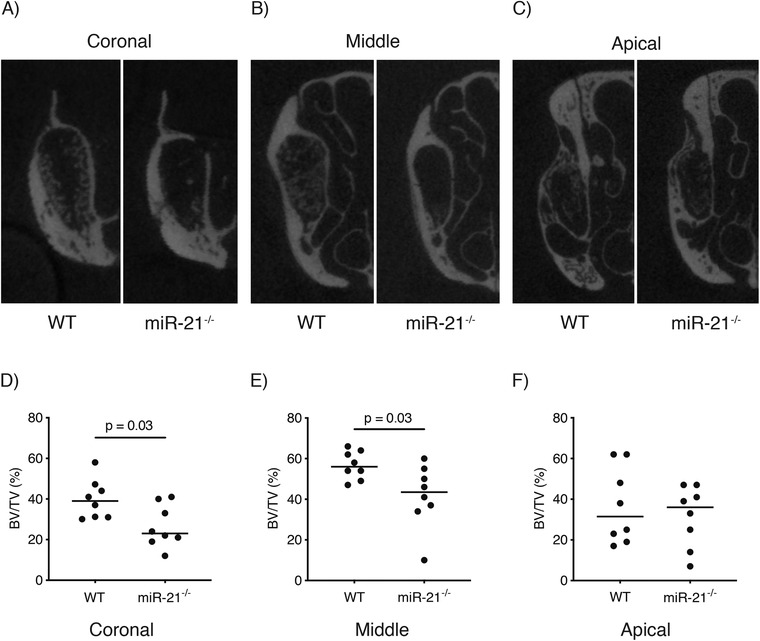
Lack of miR‐21 impairs bone regeneration of the alveolar socket. Quantitative analysis of the bone volume per tissue volume (BV/TV) in three regions of interest of the extraction socket: coronal (**A**, **D**), middle (**B**, **E**) and apical (**C**, **F**). Statistical analysis was based on Mann‐Whitney *U* test, *P* values are given

A total of two mice were excluded from the analysis because of tooth fracture; one WT and one miR‐21 knockout mouse. As a result, a total of 16 mice were analyzed. MicroCT analysis revealed that WT mice displayed higher amounts of bone volume in the coronal (Figure 2A) and middle (Figure 2B) part of the extraction socket when compared to miR‐21 knockout mice. This difference however, was not visible in the apical part of the extraction socket (Figure 2C). Quantitative analysis showed that the percentage of BV/TV at the coronal part of the extraction socket was significantly higher in WT group (Figure 2D) 39.0% (CI 31.8 to 48.0) versus 23.0% (CI 17.8 to 35.2, *P* < 0.05). Similarly, WT mice displayed significantly higher levels of BV/TV in the middle of the extraction socket (Figure 2E) when compared to the miR‐21 knockout mice, 56.0% (CI 50.9 to 62.5) versus 43.5% (CI 28.6 to 54.6, *P* < 0.05). Nevertheless, these differences were not detected in the apical part of the extraction socket (Figure 2F) 31.5% (CI 21.6 to 52.3) versus 36.0% (CI 19.0 to 44.1, *P* > 0.05).

**FIGURE 3:**
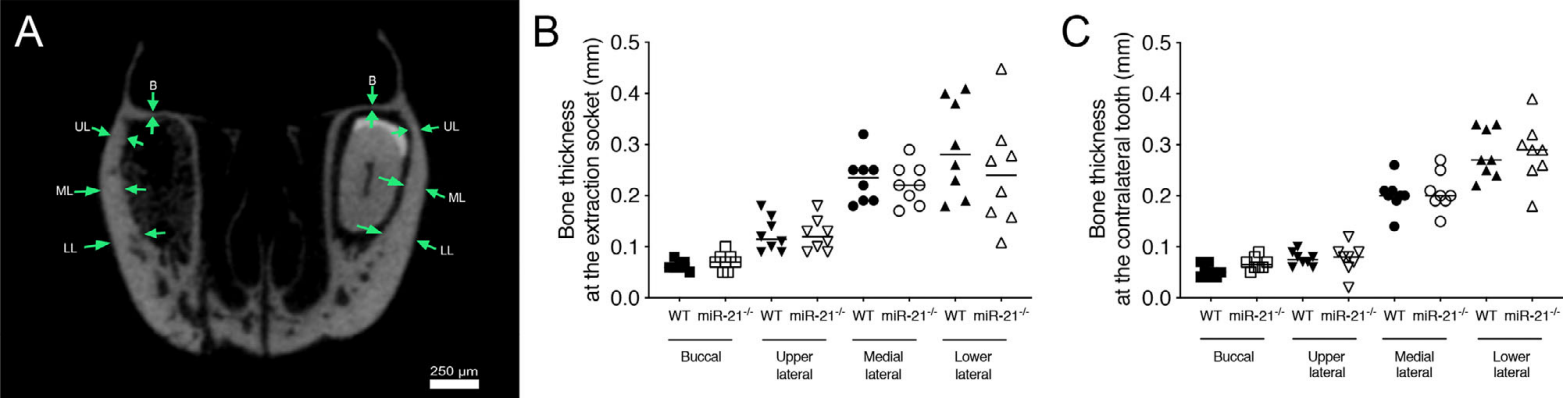
Bone thickness of the alveolar bone is not affected by miR‐21. Bone thickness of the alveolar bone at four anatomical sites (green arrows), buccal (B), upper lateral (UL), medial lateral (ML) and lower lateral (LL) (**A**). Quantitative analysis of bone thickness at the extraction socket in different anatomical sites (**B**). Quantitative analysis of bone thickness at the contralateral tooth in different anatomical sites (**C**). Statistical analysis was based on Mann‐Whitney *U* test

As shown in Figure 3A, microCT analysis further revealed that WT and miR‐21 knockout mice displayed a similar thickness of the alveolar bone in the four anatomical sites—the buccal (B), the upper lateral (UL), medial lateral (ML) and lower lateral (LL). Buccal bone thickness was comparable between both groups (Figure 3B), 0.06 mm (CI 0.06 to 0.07) in the WT and 0.07 mm (CI 0.06 to 0.09, *P* > 0.05) in miR‐21 knockout mice. Similarly, at the contralateral tooth the buccal bone thickness was 0.05 mm (CI 0.04 to 0.06) and 0.07 mm (CI 0.06 to 0.08, *P* > 0.05) (Figure 3C) in the WT and miR‐21 knockout mice, respectively. The bone thickness at the other anatomical sites; UL, ML, and LL was also similar between the groups, either in the extraction socket or in the contralateral tooth. Taken together, these findings indicate that the bone phenotype is not affected by the lack of miR‐21.

### 3.2 Histomorphometric and histological analyses

**FIGURE 4:**
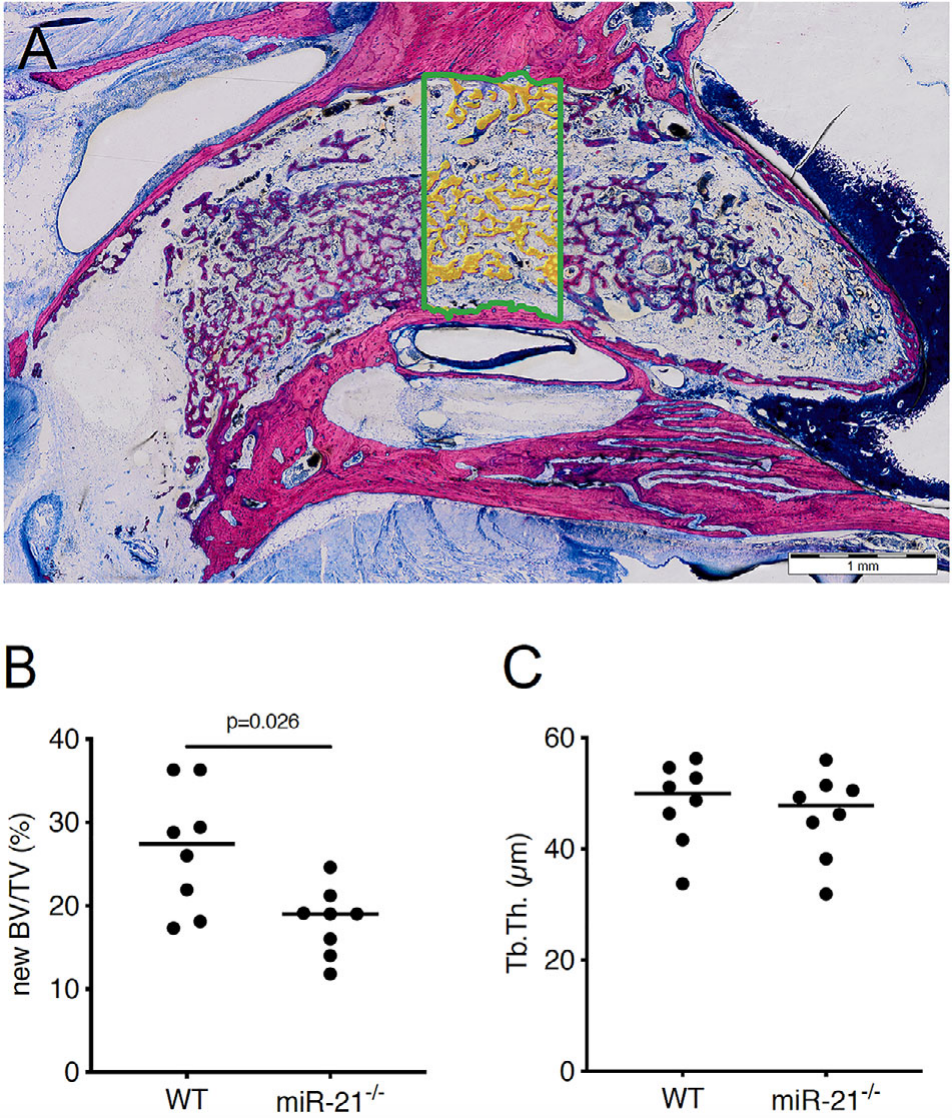
Histomorphometric analysis at the region of interest (**A**). Quantitative analysis of new bone volume per tissue volume (BV/TV) (**B**). Quantitative analysis of trabeculae thickness (Tb. Th.) (**C**). Statistical analysis was based on Mann‐Whitney *U* test, *P* values are given. Undecalcified thin ground sections stained with Levai‐Laczko

**FIGURE 5:**
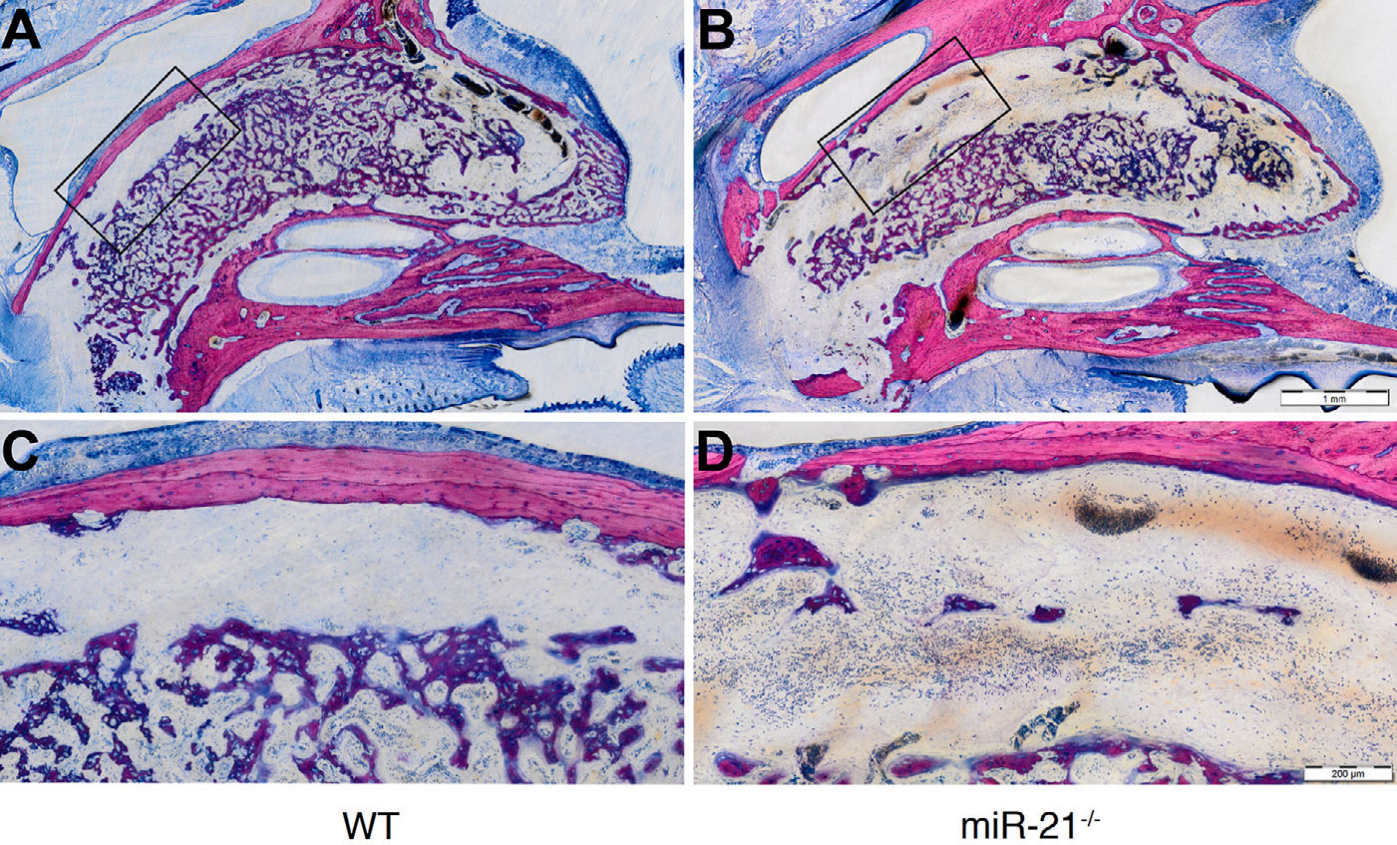
WT mice show more bone formation. Representative undecalcified thin ground sections stained with Levai‐Laczko (**A** and **B**). Region of interest at higher magnification (**C** and **D**)

**FIGURE 6:**
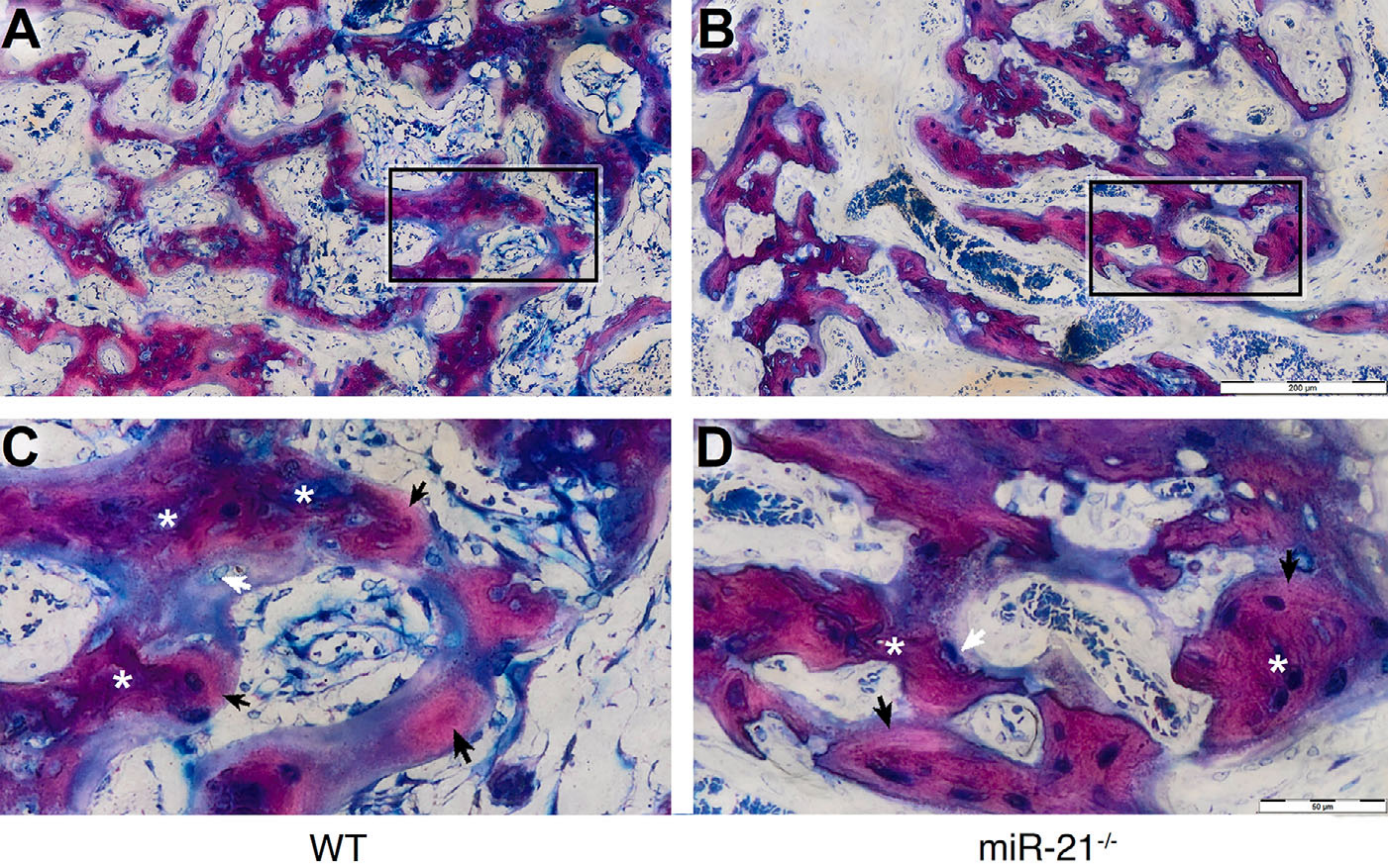
Bone features are similar between WT and miR‐21. Representative undecalcified thin ground sections stained with Levai‐Laczko (**A** and **B**). Ground sections at higher magnification (**C** and **D**). White asterisks (*) indicate woven bone, black arrows indicate parallel‐fibered bone, and white arrows indicate osteocytes getting imbedded into osteoid. Undecalcified thin ground sections stained with Levai‐Laczko

Histomorphometric analysis (Figure 4A) revealed that newBV/TV in the WT was significantly higher when compared to the miR‐21 knockout mice, 27.4% (CI 20.6 to 32.9) versus 19.0% (CI 14.7 to 21.5, *P* < 0.05) respectively (Figure 4B). The thickness of the trabeculae, nevertheless, was similar between both groups 49.9% (CI 41.9 to 54.4) versus 47.8% (CI 39.6 to 52.5, *P* > 0.05) (Figure 4C). Consistent with the microCT findings, WT mice showed more bone formation (Figure 5A) than miR‐21 knockout mice (Figure 5B). Histological analyses further revealed that in general the buccal plate remained intact (Figure 5C), showing few remodeling sites irrespective of the genetic background (Figure 5D). Moreover, the quality of the regenerated bone was rather similar between the groups (Figure 6) consisting mainly of woven bone (dark purple). This woven bone formed trabecular ridges with random orientation and was enclosed either by thin layers of parallel‐fibered bone (light purple) or thin layers of unmineralized osteoid. Unlike osteoid and parallel‐fibered bone, woven bone is rich in cells. The considerably presence of erythrocytes around the newly formed bone indicated the existence of blood vessels and a strong vascularization.

## 4 DISCUSSION

The main finding of the present study is that miR‐21 is involved in the early healing of tooth extraction sockets in murine incisors. Bone formation in the coronal and middle part of the extraction socket in miR‐21 knockout mice was around 15% lower when compared to wild type controls. These findings suggest that the lack of miR‐21 at least delays bone regeneration. It cannot, however, be concluded that miR‐21 is essential for healing of tooth extraction sockets as we did not include late time points that would represent full recovery in the wildtype mice. Nevertheless, these data support miR‐21 as a critical mechanism for alveolar socket healing suggesting a plausible molecular target to enhance bone regeneration in regenerative dentistry. This is the first study showing that a lack of miR‐21 affects bone regeneration by intramembranous bone formation. Notably, the present study also revealed that tooth extraction in mice does not cause the expected catabolic events that lead to the resorption of the buccal bone in a clinical scenario^28^ or canine models.^29^

Vieira et al. pioneered the alveolar socket healing model following tooth extraction in mice with a series of microCT, histomorphometric and molecular characterization.^8^ In that study, they showed ≈ 50% bone‐fill after 14 days, which corresponds well to our findings in the wild type mouse. In addition, the histological appearance picture of the new bone they observed resembles the one we report here—woven bone entombed by thin layers of parallel‐fibered bone. Other studies have shown that a lack of miR‐21 impairs orthodontic tooth movement^15^ and wound healing in mice^18^—even though the underlying mechanisms remain unclear. One might speculate that a lack of miR‐21 can affect osteoblast or osteoclast formation and activity, as miR‐21 is a well‐known regulator of the PTEN pathway.^30^ Moreover, miR‐21 targets genes that regulate key receptors, including bone morphogenetic protein receptor type II^31^ and TGF beta receptor II^32^ and the Wnt/β‑catenin signaling pathway thereby controlling osteogenic differentiation.^33^ Thus, miR‐21 can control major pathways involved in bone cell fate.

In support of this concept, the impaired orthodontic tooth movement^15^ might be linked to the role of miR‐21 in osteoclastogenesis, and also a possible role in osteoblast differentiation,^34^, ^35^ both being key factors for tooth movement. However, the above‐mentioned mechanisms cannot explain the impaired wound healing in mice with low miR‐21 levels.^18^ Furthermore, the inhibition of osteoclasts by bisphosphonates along with the blockade of RANKL with denosumab can boost intramembranous ossification in a fracture model^36^, ^37^ implying that the presumable impaired osteoclastogenesis related to lack of miR‐21 might also support intramembranous ossification in our tooth extraction model. One common mechanism however, that integrates wound healing and bone regeneration of defect models is angiogenesis.^38^, ^39^ Considering the importance of blood vessels for wound healing^40^ and bone regeneration,^2^ the lack of miR‐21 might impair angiogenesis thereby decreasing intramembranous ossification in our tooth extraction model.

The clinical relevance of our findings remains a matter of speculation. Nonetheless, they can be interpreted as a step towards understanding the effect of miRNAs in the healing of the alveolar socket paving the way for miRNA‐based strategies in regenerative dentistry. The preservation of the extraction socket following tooth extraction is a common procedure in daily practice, particularly in implant dentistry. Therapeutic modalities in implant dentistry may include the application of miR‐based therapeutic in combination with current regenerative approaches to counteract the alveolar bone changes following tooth extraction that may interfere the placement of dental implants. In our opinion, the current surge in genomic and proteomic data will aid in the identification of key miRs focused on miR‐targeted therapeutic approaches to support osseointegration or bone graft consolidation. Nevertheless a clear picture of miR‐21‐5p targets has yet to be drawn. miRNAs are able to target multiples pathways, in this sense it remains unknown whether the impaired socket healing is because of a defect in osteoclastogenesis, osteoblast or angiogenesis. Another limitation is that we only selected one time point. Consequently, the present study cannot represent the late and final stages of the socket healing.

## 5 CONCLUSION

These findings support the hypothesis that miR‐21‐5p is involved in the early stages of intramembranous ossification of extraction sockets in mice.

## CONFLICTS OF INTEREST

The authors declare no conflicts of interest.

## AUTHOR CONTRIBUTIONS

Reinhard Gruber and Franz Josef Strauss contributed to conception and design; contributed to acquisition, analysis, and interpretation; drafted manuscript; critically revised manuscript; gave final approval; agreed to be accountable for all aspects of work. Reiko Kobatake, Stefan Tangl, Patrick Heimel contributed to conception and design, contributed to acquisition, analysis, and interpretation; critically revised manuscript; gave final approval; agreed to be accountable for all aspects of work. Markus Schosserer, Johannes Grillari contributed to coordinating the breeding and backcrossing the miR‐21 knockout mice, as well as the selection of the experimental animals; critically revised manuscript; gave final approval; agreed to be accountable for all aspects of work. Alexandra Stahli, Matthias Hackl, Karol Ali Apaza Alaccayhuaman contributed to acquisition, analysis, and interpretation; critically revised manuscript; gave final approval; agreed to be accountable for all aspects of work.

## Supporting information

Supplementary information

## Footnotes

* AniMedica, Senden, Erlangen, Germany.

† Bayer Austria, Vienna, Austria.

‡ Leica M651, Leica Microsystems, Wetzlar, Germany.

§ HSW FINE‐JECT, Tuttlingen, Germany.

¶ Aesculap, Tuttlingen, Germany.

# Temgesic, Temgesic, Reckitt and Colman Pharm., Hull

∥ Roti‐Histofix 4%, Carl Roth, Karlsruhe, Germany.

** Scanco Medical AG, Bruttisellen, Switzerland.

†† Thermo Fisher Scientific, Waltham, MA.

‡‡ Technovit 7200 VLC + BPO; Kulzer & Co., Wehrheim, Germany.

¶¶ Exakt Apparatebau, Norderstedt, Germany.

§§ DotSlide 2.4, Olympus, Tokyo, Japan.

## Definiens, Munich, Germany.

*** Adobe, San Jose, CA.

* GraphPad Software, La Jolla, CA.
