# Supplementary material for: miRNA‐21 deficiency impairs alveolar socket healing in mice
Source: J Periodontol. 2020 Jun 20;91(12):1664–72. doi: 10.1002/JPER.19-0567 (PMC7818433; doi:10.1002/JPER.19-0567)
Supplement: Supplementary file 1 — Supplementary information [file JPER-91-1664-s001.docx]

Supplementary Video

Surgical procedure of tooth extraction in mice.

<https://drive.google.com/file/d/0B0qYie-Ebg30RU1nTEZXbzNrX3M/view>
